# Patellar tendon angle is not elevated in ACL‐injured subjects, suggesting methods to correct should focus on prehabilitation and rehabilitation rather than surgery

**DOI:** 10.1002/jeo2.12005

**Published:** 2024-02-08

**Authors:** Nicolas Cance, Michael J. Dan, Tomas Pineda, Iacopo Romandini, Guillaume Demey, David H. Dejour

**Affiliations:** ^1^ Orthopedic Surgery Department, Lyon Ortho Clinic Clinique de la Sauvegarde Lyon France; ^2^ Surgical and Orthopaedic Research Laboratory Prince of Wales Clinical School University of New South Wales Sydney New South Wales Australia; ^3^ Hospital el Carmen Santiago Chile

**Keywords:** ACL, anterior cruciate ligament reconstruction, intrinsic risk factor, patellar tendon, patellar tendon angle, tibial tubercle osteotomy

## Abstract

**Purpose:**

The aim of the study was to explore if the patellar tendon angles (PTAs) is an intrinsic risk factor for anterior cruciate ligament (ACL) rupture. We hypothesised that the PTAs will be increased in ACL rupture patients compared to matched controls.

**Methods:**

We performed a retrospective radiographic cohort study. A cohort of ACL‐injured patients between 2019 and 2022 was utilised. The control population, from the same time period, was a consecutive series of 100 patients without ligament or meniscal injuries which were prospectively added to our institutional registry. Posterior tibial slope (PTS), static anterior tibial translation (SATT), patellar tendon to tibial plateau angle (PT‐TPA), patellar tendon‐tibial shaft angle (PT‐TSA) were measured.

**Results:**

A total of 100 patients were included in the control cohort and 110 in the ACL cohort. The PT‐TPA was significantly less in the ACL cohort compared to the control cohort, mean and SD of 15.33 (±5.74) versus 13.91 (±5.68), respectively (*p* = 0.01). PT‐TSA was also less in the ACL cohort, mean and SD of 116.15 (±5.89) versus 114.27 (±4.81), however, this failed to reach statistical significance (*p* = 0.08). The PT‐TPA was not correlated with PTS (*p* = 0.65) and the PT‐TSA was inversely correlated with PTS; Pearson correlation coefficient of −0.28 (*p* < 0.01). The PT‐TSA had a greater correlation −0.4 (*p* < 0.01) with SATT than PTS 0.37 (*p* < 0.01).

**Conclusion:**

PTAs are not elevated in ACL‐injured subjects. While anteriorisation of the tibial tubercle is utilised in dogs to decrease the anterior thrust resulting from the anteriorly directed vector of the quadriceps, this treatment in the humans is not warranted and methods to reduce the PTAs should focus on prehabilitation and rehabilitation.

**Level of Evidence:**

Level III.

**Abbreviations**
ACL: anterior cruciate ligament
PT: patella tendon angle
PTS: posterior tibial slope
PT‐TPA: patellar tendon to tibial plateau angle
PT‐TSA: patellar tendon‐tibial shaft angle
SATT: static anterior tibial translation

## BACKGROUND

The anterior cruciate ligament (ACL) is responsible for controlling internal rotation and anterior translation of the femur, and is commonly ruptured in the young athlete [1].

**Figure 1:**
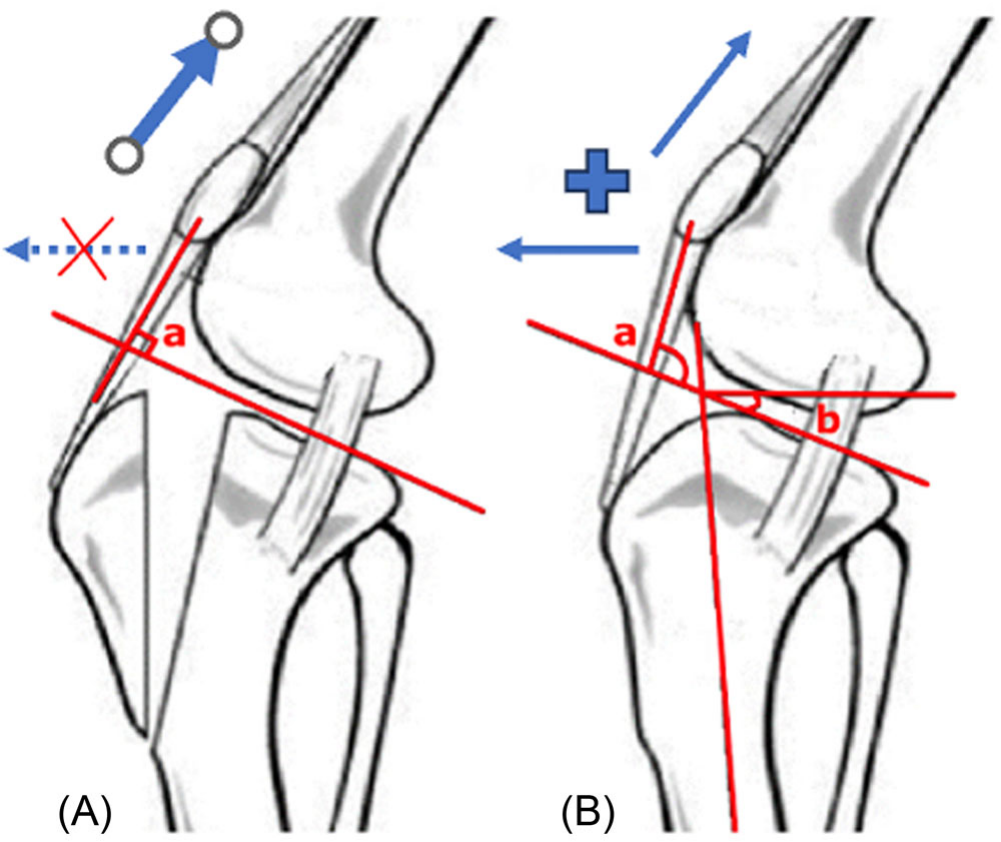
From left to right (A) TTA, (B) normal knee. In veterinary surgery the TTA aims to remove any anteriorly directed vector from the patella tendon, by making the patella tendon angle (a) perpendicular to the tibial plateau, this results in a directly compressive force with quadriceps contraction and removing any anteriorly directed translational force. By advancing the tibial tubercle anteriorly, the angle between the patella tendon and tibial plateau is reduced, this reduces the anterior component of the quadriceps vector, decreasing the stress on the ACL/CrCL. Blue arrows are the vector components for quadriceps contraction. a, patella tendon angle; ACL, anterior cruciate ligament; b, tibial plateau angle; CrCL, cranial cruciate ligament; F, femur; T, tibia; TTA, tibial tubercle advancement.

There is a naturally occurring animal model for ACL in the dog, and zoobiqity is the term used to describe the collaboration between the human and veterinary professions in order advance scientific understanding in both fields [2]. It is well known that the veterinarians had good success with tibial slope‐altering osteotomies without ACLR, to improve gait and remove anterior tibial thrust (dynamic anterior tibial translation that occurs with weightbearing). As a result this risk factor is now recognised in the human with slope‐altering osteotomies to correct this risk factor are becoming more common place [3]. One of the surgical treatments employed by Veterinarian surgeons in the treatment of ACL is a tibial tubercle advancement. By advancing the tibial tubercle, the patellar tendon angle (PTA) is reduced, removing the anterior vector of the quadriceps, with eliminates the cranial, or anterior tibial thrust with gait [4] (Figure 1).

The PTAs is the angle subtended between the axis of the tibia and the patella tendon, and reflects the anteriorly directed vector of the quadriceps [5]. In terminal extension the angle increases, which is correlated with increased ACL stress. During flexion the angle decreases along with ACL stress [6]. Following transection of the ACL the anterior tibial translation increases relative to the force and vector of the PTA.

ACL prevention exercises and rehabilitation following ACL reconstruction (ACLR) aim to decrease the patella tendon angle during landing and pivoting activities to minimise the risk of ACL rupture [7].

Individuals susceptible to ACL rupture and subsequent ACLR graft failure are known to have increased incidence of intrinsic risk factors. Such risk factors include; increased tibial slope [8], shallower medial tibial plateau [9], a narrower femoral notch [10], a more spherical‐shaped medial femoral condyle [11].

Given the PTAs are important for the mechanism of injury in a high percentage of patients, and there is clinical rationale in addressing the PTA in veterinary surgery, our aim was to explore if the PTA is an intrinsic risk factor for ACL rupture in humans.

We hypothesis that the PTAs will be increased in ACL rupture patients compared to matched controls.

## METHODS

### Ethics

All patients provided informed consent for the use of their data for research, and the study was approved by the ethical board (COS‐RGDS‐2022‐09‐008‐DEMEY‐G).

### Study design

This is a retrospective radiographic cohort study. A cohort of ACL‐injured patients between 2019 and 2022 was utilised. The control population was a consecutive series of 100 patients without ligament or meniscal injuries from the same time period, which were prospectively added to our institutional registry. We excluded patients with concomitant lateral extra‐articular procedures or meniscal injury to reduce confounders.

The inclusion criteria was lateral knee weight‐bearing radiographs at 20° of flexion. The exclusion criteria were age younger than 15 years old, or inappropriate preoperative radiographs defined as at least one of the following: no superimposed posterior femoral condyles, less than 15 cm of the proximal tibia included knee in extension or flexion more than 30° [12].

### Measurement

Radiographic measurements were performed on lateral weight‐bearing knee radiographs. The radiographs were independently reviewed and marked to calculate the measurements by two examiners (orthopaedic surgeons, M.D.). Measurements were repeated twice by each reviewer with 1‐month delay. All measurements were performed using HOROs DICOM viewer software (version 3.3.6).

**Figure 2:**
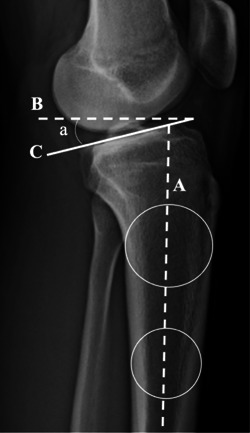
Lateral right knee radiograph demonstrating PTS. Measurement of PTS in monopodal weight‐bearing X‐rays. PTS is the angle formed between a line (B) perpendicular to the tibial diaphyseal axis (A) and the line (C) tangent to the most superior points at the anterior and posterior edges of the medial plateau. PTS, posterior tibial slope.

**Figure 3:**
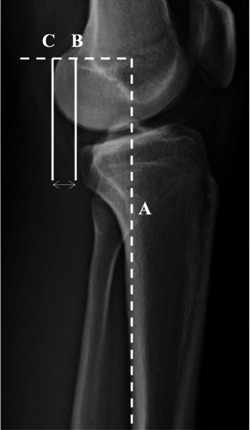
Lateral left knee radiograph demonstrating SATT. Measurement of static anterior tibial translation in monopodal weight‐bearing X‐rays. The posterior tibial cortex is the reference (line A). Two lines are traced parallel to line A and tangent to posterior part of the medial plateau (line B) and medial femoral condyle (line C). SATT is the distance between line B and C (two‐headed arrow). SATT, static anterior tibial translation.

Posterior tibial slope (PTS) (Figure 2) was measured by calculating the angle between a line perpendicular to the tibial diaphysis, and the medial tibial plateau according to Dejour et al., [13] it is used due to its improved accuracy and reliability of this technique [14]. Static anterior tibial translation (SATT) was measured as the distance between two lines parallel to the posterior tibial cortex, the first tangent to the posterior aspect of the medial tibial plateau, and the second tangent to the posterior femoral condyles [3, 13] (Figure 3).

**Figure 4:**
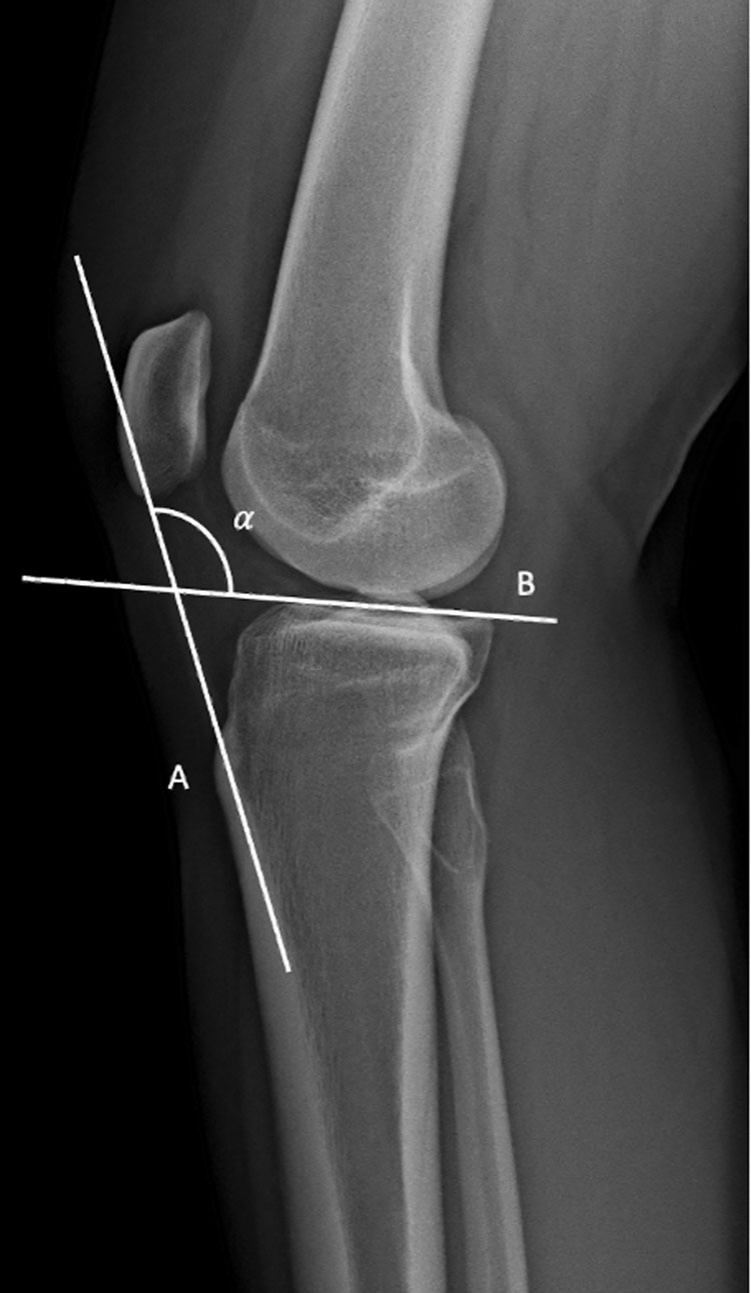
The PT‐TPA (*α*). The PTL was a line from the inferior pole of patella to the tibial tuberosity consistent with the radiolucent shadow (A). The TPL was a line extended across the medial tibial plateau, which is identified via its concave radio‐opaque line, as for PTS (B). The angle subtended between the PTL and angle TPL formed the PT‐TPA (*α*). PTL, patellar tendon line; PTS, posterior tibial slope; PT‐TPA, patellar tendon to tibial plateau angle; TPL, tibial plateau line.

We explored two PTAs. The first was the patellar tendon to tibial plateau angle (PT‐TPA) (Figure 4). The patellar tendon line (PTL) was a line from the inferior pole of patella to the tibial tuberosity consistent with the radiolucent shadow. The tibial plateau line (TPL) was a line extended across the medial tibial plateau, which is identified via its concave radio‐opaque line, as for PTS. The angle subtended between the PTL and TPL formed the PT‐TPA. This PTA was used as it is utilised in veterinary surgery [9].

**Figure 5:**
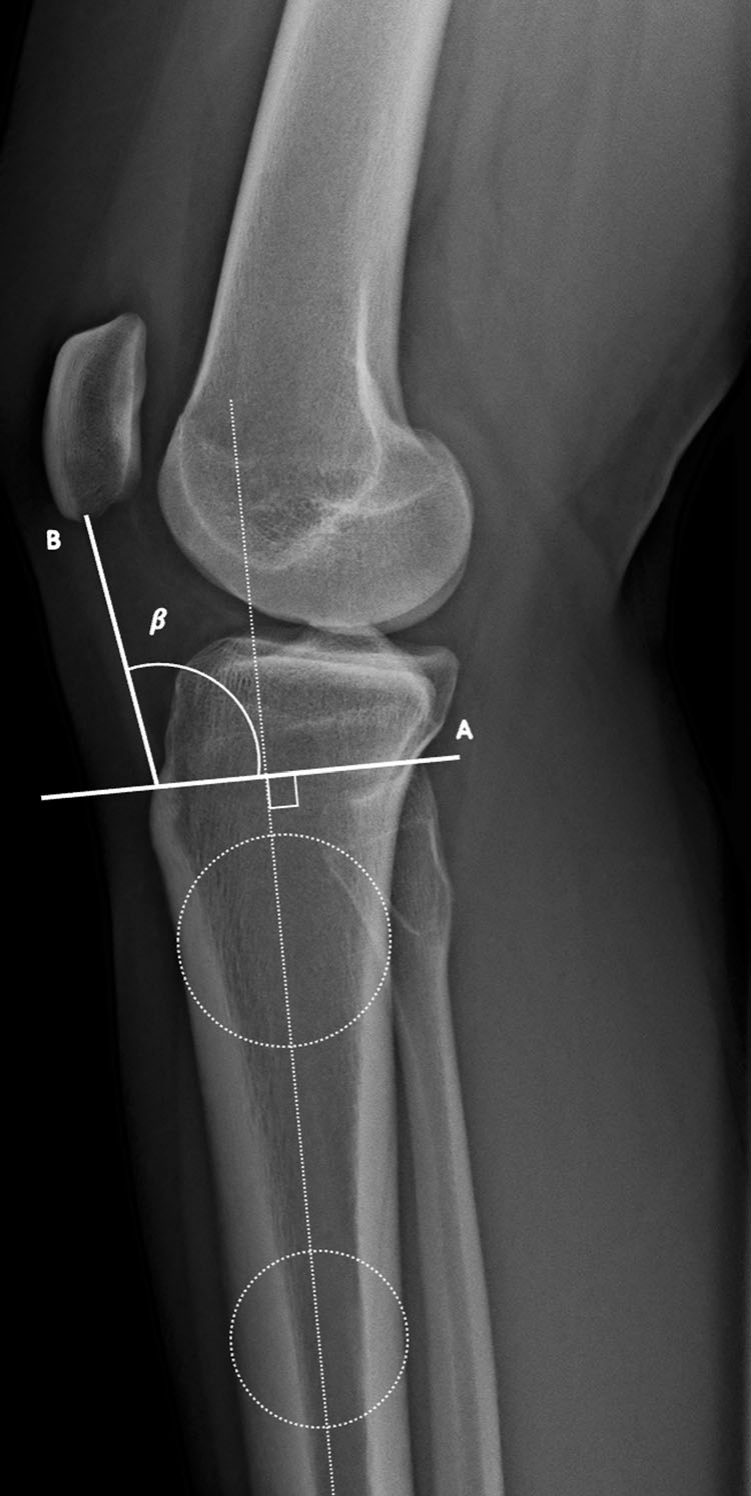
Patellar tendon‐tibial shaft angle is the angle (*β*) subtended between a line perpendicular to the tibial diaphysis (A) as described for posterior tibial slope, and the PTL (B). The PTL was a line from the inferior pole of patella to the tibial tuberosity consistent with the radiolucent shadow. PTL, patellar tendon line.

The second PTA we explored was the angle subtended between a line perpendicular to the tibial diaphysis as described for PTS, and the PTL. This formed the patellar tendon‐tibial shaft angle (PT‐ TSA). (Figure 5) This is reflective of the PTA described for the measurement of ACL force changes with applied quadriceps vectors in humans [5, 7].

### Statistical analysis

Continuous variables are expressed as the mean ± standard deviation as appropriate, while the dichotomous variables are expressed as the number and percentage of patients. The Shapiro–Wilk normality test was used to assess the normality of distributions. A two‐tailed Student's *t*‐test for independent samples was used to compare the mean values for between control and the ACL cohorts. Pearson correlation coefficient was performed to assess the correlation of gender, age, slope with the PT‐TPA and the patellar tendon‐tibial shaft angle. Univariate linear regression analysis was performed to determine the relationship between SATT and tibial slope. SPSS (v25; IBM) was used to perform these statistical analyses. Significance was set at an *α* of *p* < 0.05. With our sample size and standard deviation, a post hoc power analysis determined we were 80% powered to detect 2.2 degrees of mean difference in the PTAs between the groups.

## RESULTS

A total of 100 patients met the inclusion criteria for the control cohort and 110 for the ACL cohort. Demographics are presented in Table 1. There was a significant difference in the mean age between the cohorts (*p* < 0.001), and there was a difference in gender which approached significance (*p* = 0.097). There was no significant correlation between SATT and age (*p* = 0.263) or sex (*p* = 0.103). There was no significant correlation between PTS and age (*p* = 0.802) or sex (*p* = 0.106).

**Table 1:** Cohort comparisons.

	Control cohort	ACL cohort	*p*‐Value
Gender	Male 36.6%	Male 48.7%	0.097
Side	Right 51.5%	Right 52.2%	0.91
**Age**	**22.85 (8.04)**	**32.79 (10.78)**	**<0.001**
**PT‐TPA**	**15.33 (5.74)**	**13.91 (5.68)**	**0.01**
PT‐TSA	116.15 (5.89)	114.27 (4.81)	0.08
**SATT (mm)**	**1.27 (2.39)**	**2.33 (3.32)**	**0.04**
**PTS (°)**	**10.61 (3.28)**	**9.46 (2.85)**	**0.02**

*Note*: Gender and side reported as percentages, otherwise mean and standard deviation in brackets. *p* < 0.05 in bold.

Abbreviations: ACL, anterior cruciate ligament; PTS, posterior tibial slope; PT‐TPA, patellar tendon to tibial plateau angle; PT‐TSA, patellar tendon‐tibial shaft angle; SATT, static anterior tibial translation.

There were excellent inter‐ and intraobserver reliability of SATT measurements (intraclass correlation coefficient [ICC] = 0.99 and 0.97, respectively). There were excellent inter‐ and intraobserver reliability of PT‐TPA measurements (ICC = 0.97 and 0.94, respectively). There were excellent inter‐ and intraobserver reliability of PT‐TSA measurements (ICC = 0.95 and 0.90, respectively).

The PT‐TPA was significantly less in the ACL cohort compared to the control cohort, mean and standard deviation of 15.33 (±5.74) versus 13.91 (±5.68), respectively. PT‐TPA was also less in the ACL cohort, mean and standard deviation of 116.15 (±5.89) versus 114.27 (±4.81), however, this failed to reach statistical significance (*p* = 0.08). SATT was correlated with PTS, Pearson correlation coefficient of 0.37 (*p* < 0.01). The PT‐TPA was not correlated with tibial slope (*p* = 0.65) and the PT‐TSA was inversely correlated with tibial slope; Pearson correlation coefficient of −0.28 (*p* < 0.01). The PT‐TSA had a greater correlation −0.4 (*p* < 0.01) with anterior tibial translation than tibial slope −0.37 (*p* < 0.01) (Table 2).

**Table 2:** Pearson correlation coefficient.

	PTS	*p*‐Value	SATT	*p*‐Value
PT‐TPA	0.32	0.65	−0.22	<0.01
PT‐TSA	−0.37	<0.01	−0.4	<0.01

Abbreviations: PTS, posterior tibial slope; PT‐TPA, patellar tendon to tibial plateau angle; PT‐TSA, patellar tendon‐tibial shaft angle; SATT, static anterior tibial translation.

## DISCUSSION

The most important finding of this study was that the patella tendon angle was not increased in the ACL‐injured cohort but decreased. This was contrary to our biomechanical rationale that an increased PTAs would result in increased risk of ACL injury.

The decreased PTAs seen in ACL injury cohort is likely explained by the increased anterior tibial translation, as evidenced by the increased SATT. We demonstrated a negative correlation between PT‐TSA and both SATT and PTS, this means that with increased anterior translation, and tibial slope, the PTAs angle are decreased. Biomechanical gait studies have demonstrated this phenomenon and give a likely explanation, where there is a decrease in anterior tibial shear force occurred following ACL injury due to a decrease in the PTA resulting from the increase in anterior tibial translation [15]. Given PTS and SATT have been clinically correlated with an increased risk of ACL rupture and meniscal pathology [8, 15], and an increased PTA is associated with increased ACL strain [12], what we have likely demonstrated is post‐ACL rupture changes to the PTA that occur with weightbearing. It may be interesting to compare the contralateral limb, with an intact ACL, to confirm this phenomenon, we lacked contralateral weightbearing radiographs for comparison. This could also be achieved by comparing weightbearing and non‐weightbearing lateral radiographs to confirm this phenomenon.

The PTA is the mechanism responsible for noncontact ACL injuries. The PTA is a dynamic measure, decreasing with knee flexion and increasing with extension, resulting in increased ACL strain in terminal extension [5, 7]. This has led to prehabilitation and rehabilitation strategies to increase knee flexion with pivoting manoeuvres and landing technique [16].

The veterinary surgeons utilise osteotomies for the treatment of cranial cruciate ligament injuries, synonymous with the ACL, either utilising slope‐reducing osteotomy or tibial tubercle advancement. The goal of either osteotomy is to remove the anterior shear component with weightbearing. The slope‐reducing osteotomy, known by veterinarian's as a tibial plateau levelling osteotomy was described by veterinarian Dr. Barclay Slocum and his father, an orthopaedic surgeon. Cranial tibial thrust, defined as the cranially/anteriorly directed force produced by tibial compression during weight bearing, is responsible for cranial/anterior drawer motion in the cranial cruciate ligament deficient stifle [17]. The magnitude of the cranial tibial thrust increases (and decreases) with increase (and decrease) in the PTS angle [18]. In humans, we have also demonstrated increased PTS in those suffering recurrent ACL ruptures, and good success with slope‐reducing osteotomies, termed tibial deflexion osteotomy in the human [19, 20]. On the other hand, the tibial tubercle osteotomy reduces the PTA to remove any anteriorly directed vector with quadriceps contracture [4] (Figure 1). In humans, the Maquet osteotomy was described for patellofemoral pathology, not ACL surgery, and is rarely utilised today due to the patients nontolerance of the anteriorised bone prominence and skin necrosis [21]. The rationale from veterinarians is by advancing the tibial tubercle, the patella tendon angle is always less than 90, removing any anteriorly/cranially directed vector to the patella tendon, again, removing the cranial/anterior thrust. While the patella tendon angle has been shown to influence the amount of stress in the ACL [7], the utility of a TTA in ACL surgery for humans has not been explored until now. The results of this study suggest that further exploration of an anteriorising tibial tubercle osteotomy is not warranted in the human. There are limitations in comparing humans and dogs, dogs have a quadruped (four‐legged) gait while humans have adopted a bipedal (two‐legged) gait, this means the weight is distributed over half the limbs in the human, resulting in a elevated centre of mass, smaller base support, resulting in different knee/stifle angles during gait, which will alter the resultant PTA during stance phase [22].

We acknowledge some limitations of this study. First, it was a retrospective study from a prospective series and is therefore subjected to the limitations and biases of inherent of this design. There were differences in the age of our cohorts, and differences in gender approached significance. To account for this we confirmed that age and gender were not correlated with PTS and SATT. We also only reported radiographic outcomes and we did not correlate these findings with clinical results. However, the goal of this study was to perform an in vitro radiographic measure of translation due to axial load, which our study design allowed for. We are unaware of a previous study to compare patella tendon angles in ACL‐injured subjects to a control cohort. It may be interesting to compare the findings against the contralateral limb, with an intact ACL, and explore the change in SATT and PTA. A further limitation is we lacked contralateral weightbearing radiographs for comparison, which should be addressed in future studies. This study only explored the sagittal plane, which largely only affects anterior translation, however, the ACL is also important for rotational stability, which is unlikely to be answered by this analysis.

## CONCLUSION

The PTA is not elevated in ACL‐injured subjects. While anteriorisation of the tibial tubercle is utilised in the veterinary world to decrease the anterior thrust resulting from the anteriorly directed vector of the quadriceps, the results of this paper suggest the exploration of this treatment in the human is not warranted and methods to reduce the PTA should focus on prehabilitation and rehabilitation.

## AUTHOR CONTRIBUTIONS

*Study design, data collection, statistical analysis, literature review and manuscript writing*: Nicolas Cance. *Study design, statistical analysis, literature review and manuscript writing*: Michael Dan. *Study design, data collection, statistical analysis, literature review and manuscript editing*: Tomas Pineda. *Study design, literature review and manuscript editing*: Iacopo Romandini. *Study design, literature review and manuscript editing*: Guillaume Demey. *Study design, supervision, literature review and manuscript editing*: David H. Dejour. All authors read and approved the final manuscript.

## FUNDING

This research did not receive any specific grant from funding agencies in the public, commercial, or not‐for‐profit sectors.

## CONFLICT OF INTEREST STATEMENT

The authors declare no conflicts of interest.

## ETHICS STATEMENT

All patients provided informed consent for the use of their data for research, and the study was approved by the ethical board in advance.

## Data Availability

The datasets generated and analysed in the current study are not policy available due to data protection regulations. Access to data is limited to the researchers who have obtained permission for data processing. Further inquiries can be made to the corresponding author.
